# Ammonia signaling network: the intersection of tumor metabolism, epigenetics, and metastasis

**DOI:** 10.3389/fendo.2026.1803224

**Published:** 2026-04-27

**Authors:** Yuxin Yuan, Weisong Zhang, Xia Li, Haijue Ge, Hong Zhu

**Affiliations:** 1 The First Clinical Medical College of Nanjing Medical University, Nanjing, China; 2 Department of Thoracic Surgery, Affiliated Hospital 6 of Nantong University, Yancheng Third People’s Hospital, Yancheng, China; 3 Department of General Medicine, Affiliated Hospital 6 of Nantong University, Yancheng Third People’s Hospital, Yancheng, China; 4 Department of Gastroenterology, Affiliated Hospital 6 of Nantong University, Yancheng Third People’s Hospital, Yancheng, China

**Keywords:** ammonia signaling network, epigenetic remodeling, microenvironment, tumor metabolism, tumor metastasis

## Abstract

The ammonia signaling network plays a central role in the tumor microenvironment. It has evolved from a traditional concept of metabolic waste into a critical signaling molecule that profoundly promotes the progression of malignant tumors by regulating metabolic remodeling, epigenetic modifications, and tumor metastasis. As research continues to deepen, scientists have gradually revealed the molecular mechanisms of the ammonia signaling network in tumorigenesis and evolution, although a comprehensive understanding remains to be achieved. This network not only regulates the metabolic state of tumor cells but also enhances their migratory capacity by orchestrating epigenetic changes, thereby driving tumor metastasis and immune evasion. This article aims to systematically elucidate the significance of the ammonia signaling network in tumor metabolic dysregulation and epigenetic remodeling, delve into its role in the process of tumor metastasis, and ultimately outline potential therapeutic strategies targeting this network. By linking metabolism, epigenetics, and immunology, the ammonia signaling network provides a unified paradigm for understanding tumor biology and offers new insights and directions for future research and clinical interventions.

## 1 Introduction

For a long time, ammonia (NH3) has primarily been regarded in biology as a toxic byproduct generated during nitrogen metabolism, which the body needs to eliminate through mechanisms such as the urea cycle. However, this traditional view is being challenged in the field of tumor biology. A notable characteristic of the tumor microenvironment (TME) is the accumulation of metabolites, including ammonia. Increasing evidence suggests that the accumulation of ammonia in the TME is not merely a passive result of metabolic dysregulation, but rather a key factor actively driving malignant phenotypes (1). This shift in perspective marks the emergence of the concept of the ammonia signaling network, wherein ammonia itself acts as a signaling molecule capable of regulating various cellular biological behaviors, including metabolic reprogramming, immune modulation, and tumor progression (2). This cognitive leap from viewing ammonia as waste to recognizing it as a signal provides a novel perspective for understanding the complexity of the TME and implies that the metabolic state of tumors is not solely passively driven by oncogenic signaling pathways, but rather constitutes a dynamic, complex system feedback-regulated by the metabolites themselves.

The core role of the ammonia signaling network is reflected in its profound impact on three fundamental aspects of tumor cells. First, it directly intervenes in and reshapes the metabolic programs of tumor cells. For example, the accumulation of ammonia can activate the mammalian target of rapamycin complex 1 (mTORC1) signaling pathway, promoting the synthesis of non-essential amino acids and lipids, thereby providing a material basis for rapid cellular proliferation (3). Second, the ammonia signaling network serves as a crucial bridge connecting cellular metabolic status with the epigenetic landscape. It orchestrates patterns of histone modifications and DNA methylation by influencing the levels of key metabolites (such as S-adenosylmethionine) or directly regulating the activity of epigenetic modifying enzymes, thereby altering gene expression profiles and driving the malignant evolution of tumors (4). Finally, these ammonia-driven metabolic and epigenetic changes ultimately converge to enhance the malignancy of tumors, particularly in terms of metastasis and immune evasion. Ammonia can promote epithelial-mesenchymal transition (EMT), endowing tumor cells with migratory and invasive capabilities (5). At the same time, it creates an immunosuppressive niche for the dissemination and survival of tumor cells by directly inhibiting the function of cytotoxic immune cells such as natural killer (NK) cells and T cells (1, 6).

In light of the emerging and central role of the ammonia signaling network in tumor biology, this review aims to comprehensively and systematically outline the latest advancements in this field. The article will begin with the biological foundation of the ammonia signaling network, detailing its molecular composition and regulatory mechanisms. Subsequently, it will delve into the specific roles of this network in driving tumor metabolic reprogramming, epigenetic remodeling, tumor metastasis, and immune evasion. Building upon this foundation, the article will further discuss the clinical significance of the ammonia signaling network, including its potential as a biomarker and strategies for therapeutic targeting. Finally, the article will look ahead to future research directions and challenges, aiming to provide theoretical references and forward-looking guidance for in-depth exploration and clinical translation in this field.

## 2 The biological structure of ammonia signaling networks

### 2.1 Sources and metabolic fate of ammonia in the TME

The accumulation of ammonia in the TME is the result of various metabolic pathway abnormalities. Its diverse sources collectively contribute to a localized environment with high ammonia levels.

Glutamine catabolism is the primary pathway for ammonia production in tumor cells. In many rapidly proliferating malignant tumors, cells exhibit a form of addiction to glutamine (7). Glutamine is catalyzed by glutaminase (GLS) to release an amide group, generating glutamate and ammonia (8). This process not only provides carbon skeletons for the TCA cycle but also releases a significant amount of ammonia. Clinical data indicate that high expression of GLS is closely associated with poor prognosis in patients with various tumors, such as breast cancer and lung cancer, highlighting the importance of this pathway in tumor progression (9). To meet the survival and proliferation demands in nutrient-limited environments, tumor cells enhance protein catabolism. Protein degradation mediated by the ubiquitin-proteasome system and autophage release various amino acids and their metabolic products, including ammonia (10). This method of obtaining nutrients by degrading endogenous or engulfed proteins not only provides energy and biosynthetic precursors to the cells but also exacerbates the accumulation of ammonia in the microenvironment (11). In hepatocellular carcinoma (HCC), dysfunction of the urea cycle is a specific and critical mechanism leading to ammonia accumulation. The urea cycle is the primary pathway for the body to eliminate ammonia. Studies have found that the loss of the tumor suppressor p53 leads to downregulation of critical enzymes in the urea cycle, such as carbamoyl phosphate synthetase 1 (CPS1) and ornithine transcarbamylase (OTC), severely impairing the ability of liver cancer cells to clear ammonia, resulting in a dramatic increase in intracellular ammonia concentration (12–14). This makes the HCC microenvironment a typical model for studying ammonia signaling. In addition to the endogenous production of ammonia by tumor cells, the gut microbiome is also an important source of systemic ammonia. Gut bacteria generate a significant amount of ammonia by decomposing dietary proteins and urea, which then enters the liver via the portal venous system (5). While the systemic impact of gut-derived ammonia is well-established in models of hepatic encephalopathy, direct *in vivo* evidence demonstrating its influence on distant solid tumor microenvironments via systemic circulation remains limited and largely speculative. However, the spatial proximity of the microbiome plays a definitive role in localized tumors. In primary gastrointestinal malignancies, such as colorectal cancer (CRC), robust experimental evidence indicates that microbiota-derived ammonia accumulates directly within the local TME, where it actively drives T cell exhaustion and enhances tumor progression (11). This underscores the notion that the contribution of the gut microbiome to the ammonia signaling network is highly dependent on anatomical localization and local diffusion.

### 2.2 Molecular components: transport proteins, sensors, and effectors

The operation of the ammonia signaling network relies on a series of key molecular components responsible for the transmembrane transport of ammonia, the sensing of intracellular concentrations, and the transmission of downstream signals. The Rh protein family (e.g., RhBG) serves as crucial transport proteins mediating ammonia-facilitated diffusion. They not only regulate the transmembrane rate of ammonia but also directly participate in signal transduction. For instance, RhBG can interact with myeloid differentiation primary response protein 88 (MyD88), initiating downstream signaling cascades that ultimately activate the nuclear factor kappa-light-chain-enhancer of activated B cells (NF-κB) pathway (15). This discovery directly links the transport process of ammonia to inflammatory responses and cell survival signals, revealing the function of transport proteins themselves as signal integration platforms.

Recent studies have proposed a groundbreaking revision regarding the role of Solute Carrier Family 4 Member 11 (SLC4A11) in tumor ammonia metabolism. The earlier viewpoint suggested that, given the cytotoxicity of ammonia, SLC4A11 might function as an ammonia efflux pump, aiding tumor cells in expelling excess ammonia; thus, its inhibition could trap ammonia within cells to exert toxic effects. However, recent evidence from high-impact studies clearly indicates that, in HCC, SLC4A11 actually plays a role in ammonia influx, particularly within the cancer stem cell (CSC) subpopulation. Mechanistically, the expression and function of SLC4A11 are tightly regulated at multiple levels. At the transcriptional level, the aberrant activation of the Wnt/β-catenin signaling pathway directly upregulates SLC4A11 expression, facilitating ammonia scavenging (16). At the post-translational and structural levels, the transport activity of SLC4A11 is allosterically regulated by specific membrane lipids. For instance, the binding of phosphatidylinositol 4,5-bisphosphate (PIP2) stabilizes SLC4A11 in an active, outward-facing conformation, which is essential for efficient ammonia influx (17). The SLC4A11-mediated ammonia influx provides a crucial nitrogen source for the *de novo* synthesis of amino acids and nucleotides in CSCs, thereby promoting their stemness maintenance and tumor-initiating capacity. Consequently, the high expression of SLC4A11 is closely associated with poor prognosis in HCC, and inhibiting its function, that is, blocking ammonia influx, has emerged as a targeted therapeutic strategy against HCC stem cells (16, 18, 19). This shift in understanding reveals the astonishing plasticity of tumor cells under metabolic stress, transforming a potential toxin into a key nutrient.

mTORC1 is a core kinase that enables cells to sense nutrients and regulate anabolic metabolism. The accumulation of ammonia can promote the synthesis of non-essential amino acids, and amino acids are potent activators of mTORC1 (20). Therefore, a high-ammonia environment continuously activates the mTORC1 signaling pathway by providing ample amino acids, thereby enhancing protein synthesis, lipid generation, and inhibiting autophagy, which provides a robust impetus for the growth and proliferation of tumor cells (21). Building upon this classical understanding, recent research has elucidated that the ammonia-mTORC1 axis involves a highly dynamic and spatially regulated interplay at the lysosomal surface. Glutamine-derived ammonium actively modulates lysosomal pH, optimizing its degradation capacity and sustaining mTORC1 activation during early nutrient starvation (22). Moreover, under nutrient-deprived conditions typical of the TME, cancer cells adopt alternative nutrient acquisition strategies such as macropinocytosis. In this context, specific lysosomal transporters (e.g., SNAT7) facilitate the export of amino acids derived from engulfed extracellular proteins, thereby driving robust, Rag GTPase-independent mTORC1 activation (23). Notably, this network is highly context-dependent; for instance, in β-catenin-mutated liver cancers, impaired ammonia clearance exacerbates hyperammonemia, leading to a compensatory accumulation of glutamate-derived amino acids that paradoxically hyperactivates mTORC1 (24). Hypoxia-inducible factor 1α (HIF-1α) is a key transcription factor that allows cells to respond to hypoxic environments. The accumulation of ammonia, particularly under the hypoxic conditions commonly found in tumors, can enhance the stability and activity of HIF-1α. This further drives the shift of cells towards a glycolytic metabolic pattern and upregulates the expression of a series of genes associated with angiogenesis, cell survival, and invasion, thereby synergistically promoting the malignant progression of tumors (25, 26).

**Figure 1:**
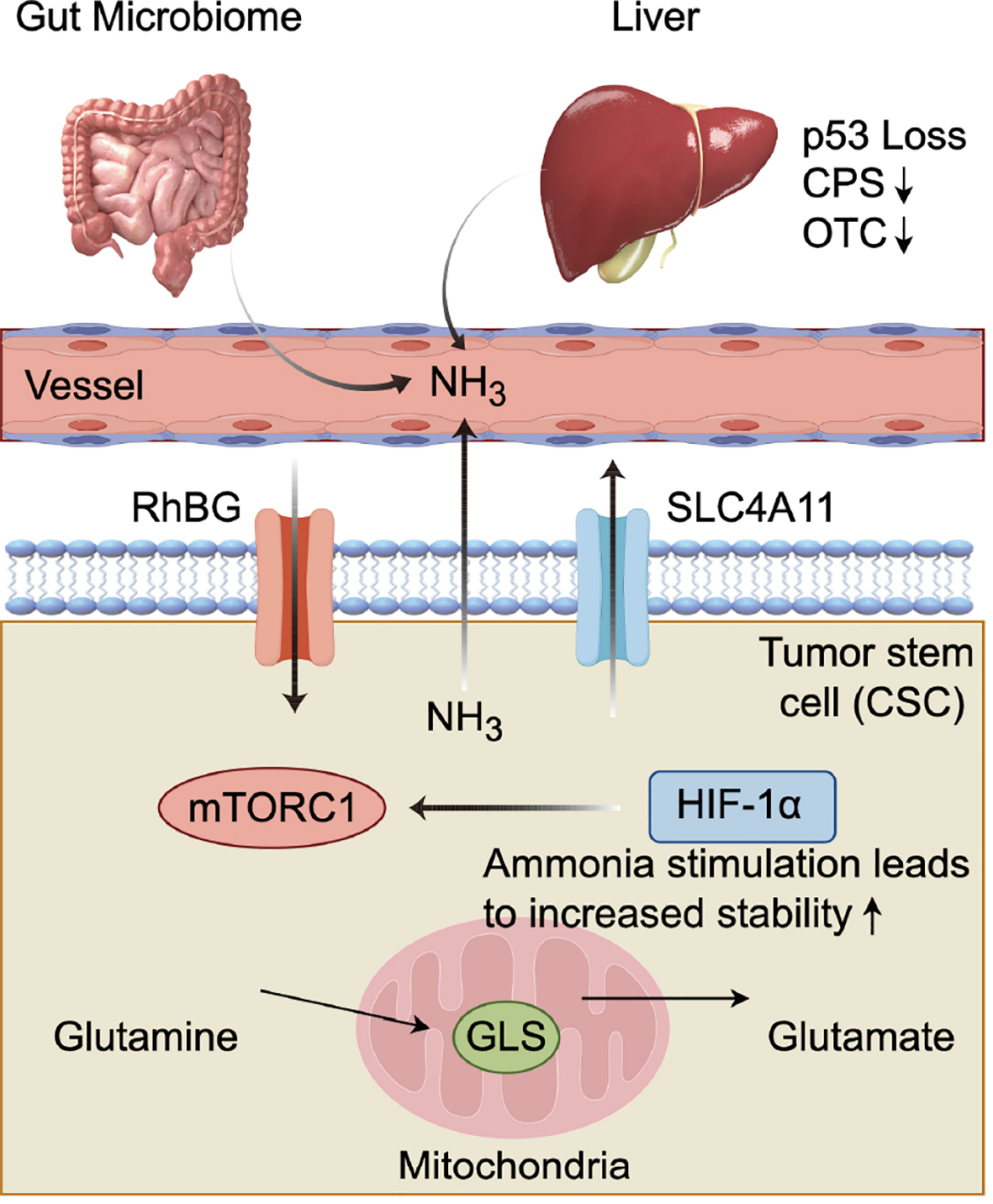
Sources, transport, and signal transduction of ammonia signaling networks in the TME.

These molecular components together form a precise regulatory circuit. For instance, ammonia produced from glutamine metabolism is sensed and regulated by transport proteins such as RhBG and SLC4A11. The high intracellular concentration of ammonia activates mTORC1, issuing a synthesis signal; simultaneously, it stabilizes HIF-1α, signaling adaptation to hypoxia and alteration of energy metabolism. This integrated system enables tumor cells to tightly couple the availability of nitrogen sources (via ammonia) with growth signals (mTORC1) and energy metabolism strategies (HIF-1α), thereby achieving robust proliferation in a challenging microenvironment. This networked regulatory pattern also suggests that merely targeting a single node (such as GLS) may have limited efficacy, as the network possesses compensatory capabilities, and employing a multi-target combined intervention strategy may be more effective (**Figure 1**). We summarize the key molecules in the ammonia signaling network and their roles in tumor progression (**Table 1**).

**Table 1:** Key molecules in the ammonia signaling network in tumor progression.

Molecule	Category	Main function	Role in tumor
Glutaminase	Ammonia source enzyme	Catalyzes the conversion of glutamine to glutamate and ammonia	Provides nitrogen and carbon sources for tumors, major source of ammonia
CPS1, OTC	Ammonia metabolic enzymes	Key enzymes in the urea cycle responsible for ammonia clearance	Often downregulated in HCC due to p53 loss, leading to ammonia accumulation
RhBG	Ammonia transporter protein	Facilitates ammonia transmembrane transport; signal transduction	Regulates intracellular ammonia concentration; activates NF-κB pathway via MyD88
SLC4A11	Ammonia transporter protein	Facilitates ammonia influx in HCC cancer stem cells	Supports biosynthesis and stemness of cancer stem cells
mTORC1	Signaling kinase	Integrates nutrient signals to regulate cell growth and anabolic metabolism	Activated by ammonia, promotes protein and lipid synthesis, inhibits autophagy
HIF-1α	Transcription factor	Regulates cellular adaptation to hypoxic environments	Stabilized by ammonia, promotes glycolysis and angiogenesis

## 3 Ammonia signal-driven metabolic reprogramming

The ammonia signaling network forcibly reprograms cells into a metabolic phenotype that adapts to tumor growth and metastasis by interfering with core intracellular metabolic pathways. This reprogramming is not only aimed at meeting energy demands but also at generating specific metabolites and signals to support malignant behaviors.

### 3.1 Disruption of central carbon metabolism: glycolysis and the TCA cycle

The regulatory role of ammonia in central carbon metabolism is central to its driving force for metabolic reprogramming. Studies have confirmed that high concentrations of ammonia specifically inhibit alpha-ketoglutarate dehydrogenase (α-KGDH), a key rate-limiting enzyme in the TCA cycle. The inhibition of α-KGDH decreases the flux through the TCA cycle and significantly impairs oxidative phosphorylation. To compensate for this energy deficit, cells are compelled to enhance glycolytic activity, resulting in the conversion of substantial amounts of glucose into lactate, even in the presence of adequate oxygen. This phenomenon is referred to as the intensification of the Warburg effect (27, 28). This shift in metabolic pattern has profound consequences. Firstly, the accumulation of lactate leads to acidification of the TME, which not only aids tumor cells in degrading the extracellular matrix and enhancing their invasive capacity but also suppresses the function of immune cells (29). Secondly, the blockage of the TCA cycle results in the accumulation of upstream metabolites, such as elevated levels of specific amino acids (e.g., aspartate) (30). These accumulated amino acids function as signaling molecules rather than merely serving as biosynthetic building blocks. They initiate specific cascades that activate the translation of invasive proteins, thereby significantly enhancing the metastatic potential and survival of tumor cells (31). Therefore, the metabolic phenotype induced by ammonia is a carefully orchestrated package deal: it sacrifices the energy efficiency of oxidative phosphorylation in exchange for rapid ATP supply, ample biosynthetic precursors, and the production of signaling metabolites that can directly drive malignant progression.

### 3.2 Reprogramming of lipid metabolism

In addition to carbohydrate metabolism, the reprogramming of lipid metabolism is a crucial aspect of tumor cells adaptation to the demands of rapid proliferation, with ammonia playing an important driving role in this process. The accumulation of ammonia can activate Sterol Regulatory Element Binding Protein 1 (SREBP-1), which is the core transcription factor regulating the expression of genes related to lipogenesis and fatty acid metabolism (32). This regulatory network is particularly evident in HCC. The aberrant activation of the classical Wnt/β-catenin signaling pathway not only promotes the transcription of genes encoding ammonia-metabolizing enzymes, thereby increasing ammonia production, but also enhances the efficiency of cellular utilization of ammonia by upregulating the expression of Glutamate Dehydrogenase (GLUD1). This process forms a positive feedback loop: β-catenin activation → increased ammonia production and utilization → SREBP-1 activation → enhanced fatty acid synthesis. The newly synthesized fatty acids provide essential materials for rapidly dividing tumor cells to construct new cell membranes and may also act as signaling molecules involved in other biological processes, collectively supporting tumor growth and development (33, 34).

### 3.3 Ammonia-induced mitochondrial dysfunction

Mitochondria, as the energy hub and metabolic center of cells, are one of the primary targets of ammonia toxicity. High concentrations of ammonia can cause significant damage to mitochondrial function. Much of the foundational evidence regarding ammonia-induced mitochondrial dysfunction, specifically the direct inhibition of respiratory chain complex IV and the subsequent severe oxidative stress, has primarily been characterized in non-tumor neurotoxic models, such as hepatic encephalopathy. While these non-tumor models clearly establish the biochemical basis of ammonia toxicity, the exact magnitude of these effects within the highly resilient and metabolically reprogrammed mitochondrial networks of tumor cells necessitates further direct validation. Nevertheless, extrapolating to the TME suggests that damaged electron transport chains may leak electrons that react with oxygen molecules, generating superoxide and other reactive oxygen species (ROS), which in turn trigger oxidative stress within the cell (35, 36). This state of oxidative stress itself serves as a pro-cancer signal, capable of activating various pro-survival and pro-metastatic signaling pathways. Additionally, ammonia may interfere with the β-oxidation of fatty acids by modulating signaling pathways within the mitochondria, leading to further imbalances in lipid metabolism. Therefore, ammonia-induced mitochondrial dysfunction is not only a direct cause of cellular energy metabolism disorders but also a critical node linking ammonia signaling with oxidative stress, lipid metabolism abnormalities, and multiple pathological processes (37–39).

### 3.4 The dual nature of ammonia: concentration-dependent toxicity

While the aforementioned mechanisms highlight the pro-tumorigenic roles of the ammonia signaling network, it is crucial to delineate its boundaries. Emerging metabolic paradigms suggest that ammonia exerts a highly concentration-dependent, biphasic effect, potentially manifesting tumor-suppressive properties under specific microenvironmental conditions. At excessively high, unbuffered concentrations, ammonia transitions from being a signaling nutrient to a potent cytotoxin. It induces severe osmotic stress, profound mitochondrial depolarization, and extensive oxidative damage, ultimately leading to apoptosis or necrosis, even in malignant cells. Foundational studies have demonstrated that while cancer cells recycle low levels of ammonia to support biomass, the accumulation of ammonia beyond a critical threshold becomes lethal if the cells lack robust ammonia assimilation and clearance mechanisms, such as sufficient expression of glutamine synthetase (40). Consequently, in poorly adapted tumor subclones or in highly enclosed microenvironments where diffusion is restricted, localized over-accumulation of ammonia presents a significant barrier to tumor progression. This dual nature underscores the necessity for therapeutic strategies to be highly context-dependent.

## 4 Ammonia signaling network orchestration and epigenetic remodeling

Ammonia serves as a crucial link between cellular metabolic states and gene expression regulation, reshaping the epigenetic landscape of tumor cells through direct or indirect mechanisms. It demonstrates how a simple metabolite can “write” or “erase” the epigenetic marks of cells, profoundly altering their biological behavior.

### 4.1 The impact of ammonia on the histone code

Post-translational modifications of histones, commonly referred to as the histone code, are critical mechanisms for regulating chromatin structure and gene transcription. Ammonia can interfere with the reading and writing processes of this code in various ways. Research indicates that high concentrations of ammonia can inhibit the activity of histone demethylases (KDMs). KDMs are responsible for removing methylation marks from histones, and their functional suppression leads to an abnormal increase in the methylation levels of histones, particularly at certain key sites (such as H3K4, H3K9, H3K27, etc.), resulting in a hypermethylated state. This state alters the conformation of chromatin, disrupting the binding of transcription factors to DNA, thereby inhibiting the expression of tumor suppressor genes or aberrantly activating oncogenes, ultimately affecting the proliferation, differentiation, and migration abilities of cells (41). In contrast to methylation, ammonia's effect on histone acetylation is primarily promotive. It can enhance the acetylation level of histone H3 at lysine 27 (H3K27ac). H3K27ac is a key marker of active enhancers and promoters, and its increased level is usually directly associated with the activation of gene transcription. By elevating H3K27ac levels, ammonia can enhance the transcriptional activity of a range of key genes associated with tumor growth, proliferation, and metabolic reprogramming. This process may be related to ammonia s activation of specific signaling pathways (such as the MAPK pathway), which can further activate histone acetyltransferases (HATs), thereby translating metabolic signals into epigenetic activation marks (42–44).

### 4.2 Disruption of the DNA methylation landscape

**Figure 2:**
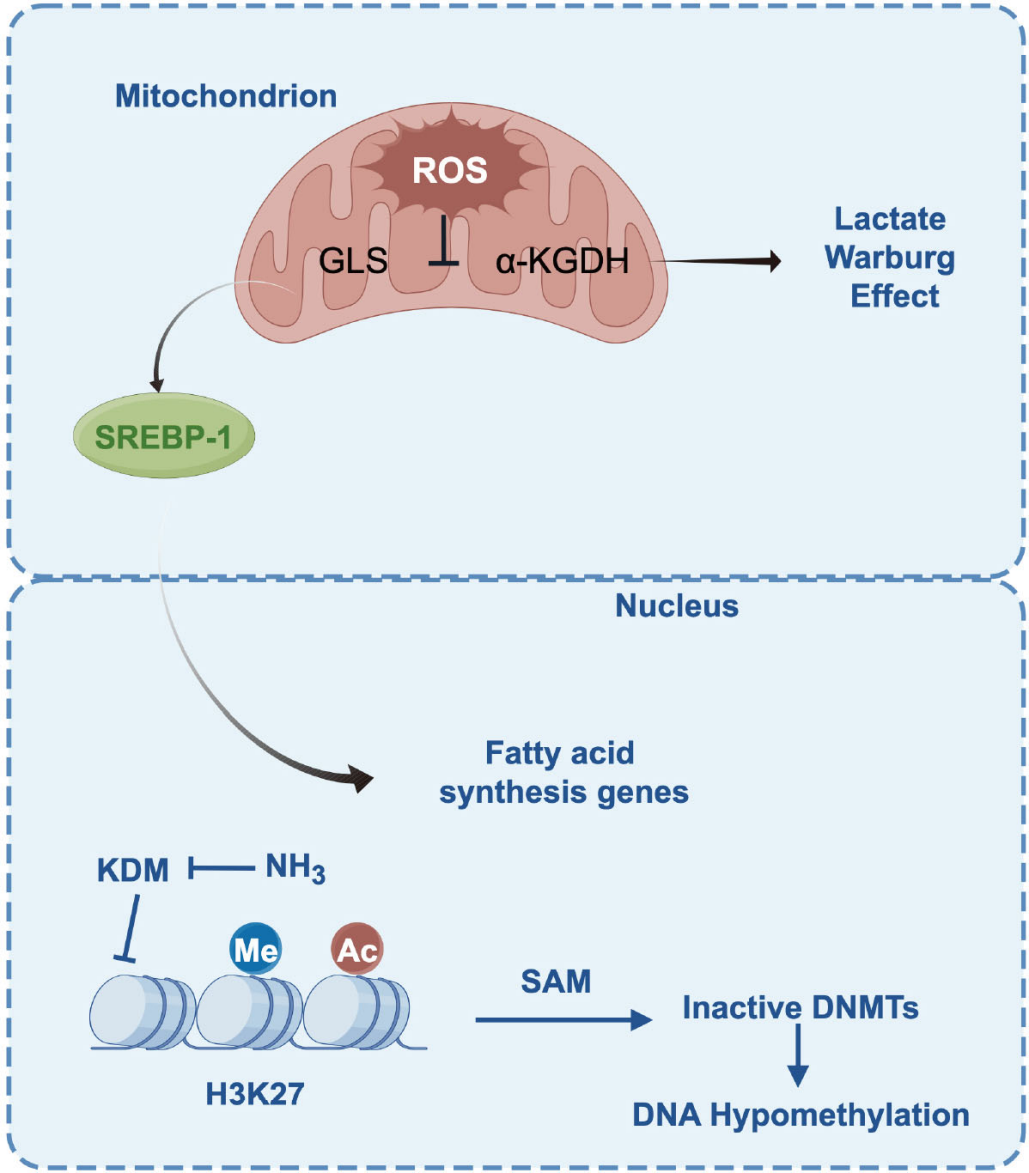
Ammonia-induced mitochondrial dysfunction, metabolic flux rerouting, and epigenetic reprogramming.

DNA methylation is another crucial epigenetic regulatory mechanism, and ammonia can profoundly impact it by interfering with the “methylation economy” within cells. The SAM-DNMT axis: The core of this mechanism lies in S-adenosylmethionine (SAM), which serves as the universal methyl donor for nearly all methylation reactions in cells, including DNA methylation. The metabolic process of ammonia, particularly its conversion to glutamine or its involvement in the synthesis of other amino acids, requires substantial energy and metabolic intermediates, indirectly leading to an increased burden on the SAM biosynthetic pathway and the depletion of the SAM pool. A decrease in SAM concentration directly weakens the activity of DNA methyltransferases (DNMTs), as the catalytic reactions of DNMTs are highly dependent on SAM as a substrate (45–48). The reduction in DNMT activity results in a decrease in DNA methylation levels across the genome, leading to global hypomethylation. This hypomethylated state particularly affects gene regions that are normally silenced through methylation in healthy cells, including many oncogene promoters and regulatory elements. For instance, studies have shown that following ammonia treatment, the methylation levels of the promoter regions of oncogenes such as glutamine synthetase (GLUL) are significantly reduced, resulting in their abnormal upregulation and promoting tumor growth (49). Furthermore, global hypomethylation may further enhance the growth and metastatic capabilities of tumor cells by altering the binding patterns of transcription factors and the accessibility of chromatin. The direct biochemical link between ammonia-mediated metabolism and epigenetics provides a solid theoretical foundation for developing metabolism-epigenetics combination therapeutic strategies. For instance, in tumors with elevated ammonia levels, the use of glutaminase inhibitors to reduce ammonia production may restore normal intracellular levels of SAM and DNMT activity, potentially rendering tumor cells sensitive again to epigenetic drugs such as DNMT inhibitors (**Figure 2**).

## 5 The role of ammonia signaling in tumor metastasis and immune evasion

The ammonia signaling network concurrently influences two critical processes in dangerous tumor metastasis: it inherently boosts the mobility and invasion potential of tumor cells, while also undermining the immune defense that is supposed to eradicate these spreading cells, thus promoting their widespread dissemination.

### 5.1 Promotion of epithelial-mesenchymal transition

EMT is a crucial biological process through which epithelial-derived tumor cells acquire migratory and invasive capabilities, characterized by the loss of cell polarity and tight junctions, transitioning to a morphology with mesenchymal features. Ammonia is a potent inducer of the EMT process. The Transforming Growth Factor-beta (TGF-β) signaling pathway is a classical mechanism driving EMT. Research has demonstrated that ammonia exposure significantly activates the TGF-β/Smad pathway. Notably, classical *in vivo* studies of ammonia-induced pathological EMT have predominantly employed non-tumor models. For example, in environmental exposure models using both chicken and mouse subjects, ammonia exposure results in pathological EMT characteristics in tissues, including the downregulation of the epithelial marker E-cadherin, upregulation of the mesenchymal marker vimentin, disruption of intercellular junctions, and tissue fibrosis. While direct *in vivo* validation within the primary TME is still developing, these highly conserved tissue remodeling responses strongly suggest a parallel mechanism in malignant progression (50, 51). The activation of the TGF-β signaling pathway ultimately converges on the upregulation of a series of core EMT transcription factors (EMT-TFs), including Snail, Twist, and members of the ZEB family (ZEB1 and ZEB2). These transcription factors are the orchestrators of the EMT program; they directly bind to the promoters of epithelial genes such as E-cadherin, inhibiting their transcription while simultaneously activating the expression of mesenchymal genes. Research indicates that ammonia dependency can lead to elevated expression levels of ZEB1 and ZEB2, closely associated with enhanced cell migratory capacity. Therefore, ammonia effectively initiates the EMT program by activating the TGF-β → ZEB1/2 axis, providing a molecular basis for local invasion and distant metastasis of tumor cells (52, 53).

### 5.2 Creating an immunosuppressive microenvironment

Tumor cells must evade the surveillance and attack of the host immune system during the metastatic process. Ammonia systematically weakens the anti-tumor immune response through various mechanisms, creating an immunosuppressive microenvironment conducive to tumor survival and dissemination. NK cells and cytotoxic T lymphocytes (CTLs) are the backbone of anti-tumor immunity, as they kill tumor cells by releasing perforin and granzymes. Research has clearly indicated that the accumulation of ammonia interferes with the storage and release of mature perforin within these cells, thereby directly reducing their cytotoxic activity and impairing their ability to eliminate tumor cells. Further studies have unveiled a more direct killing mechanism: within activated CD8+ T cells, the gradual accumulation of intracellular ammonia induces damage to lysosomes and mitochondria, ultimately leading to programmed cell death of T cells. This explains why T cells often exhibit functional exhaustion and even a reduction in number within high-ammonia tumor microenvironments (54, 55).

**Figure 3:**
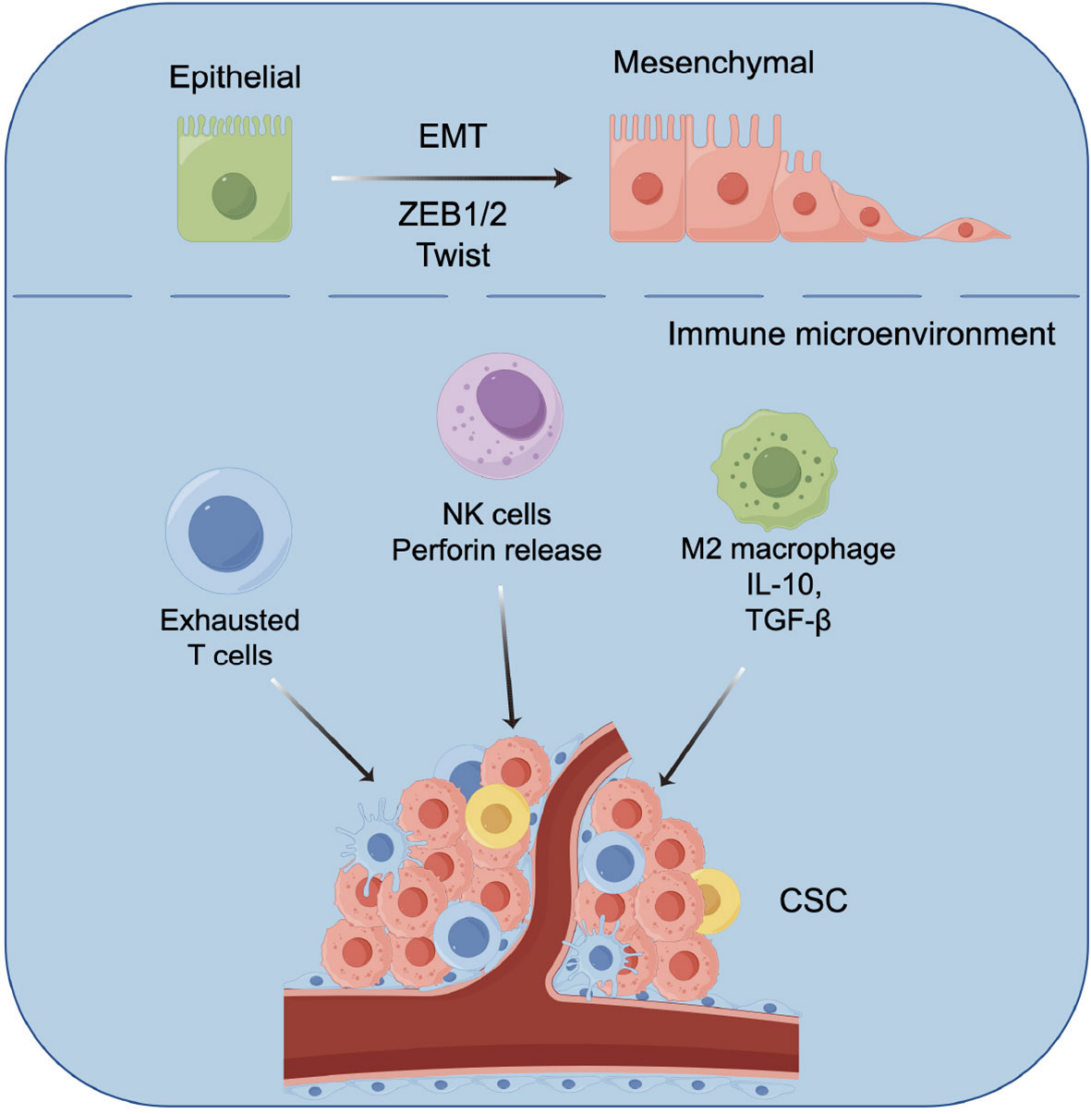
The ammonia signaling network synergistically promotes the EMT and the formation of an immunosuppressive microenvironment.

Tumor-associated macrophages (TAMs) are among the most abundant immune cells in the TME, exhibiting highly plastic functions. TAMs can be polarized into M1 type with anti-tumor activity or M2 type that promotes tumor growth and immune suppression. A high concentration of ammonia can strongly drive macrophage polarization towards the M2 type. M2 macrophages further inhibit T cell activity by releasing various immunosuppressive factors such as interleukin-10 (IL-10) and TGF-β, promoting angiogenesis and tissue remodeling, thereby supporting tumor growth and metastasis. In summary, ammonia plays a coordinating role in the TME; it not only initiates the escape program (EMT) of tumor cells but also disarms the alarm system of the immune system (suppressing NK/T cells and inducing M2 macrophages). This synergistic effect suggests that therapies targeting ammonia metabolism possess dual potential: they may inhibit the metastatic capability of tumor cells while also restoring anti-tumor immune responses, providing a new strategy for converting cold tumors into hot tumors that are sensitive to immunotherapy (56) (**Figure 3**).

## 6 Interaction with other stress response networks

The processes of tumor metastasis and immune evasion discussed in the previous section are highly complex biological events that do not occur in isolation. The ammonia signaling network that drives these malignant behaviors is intricately linked with other essential intracellular stress response networks, particularly those involving ROS and hypoxia (HIF-1α) signaling. These stress signals serve as critical amplifiers for ammonia-mediated functions. For instance, hypoxia-induced HIF-1α synergizes with ammonia to further upregulate EMT transcription factors, facilitating metastasis. Concurrently, ROS accumulation influences the polarization of immunosuppressive macrophages, aiding in immune evasion. This intricate interaction establishes a robust, self-reinforcing adaptive network that significantly enhances the survival and resilience of tumor cells within a challenging microenvironment.

### 6.1 Ammonia-ROS positive feedback loop

Oxidative stress is a state caused by the imbalance between the production of ROS and the cellular antioxidant defense system, playing a dual role in tumorigenesis and progression. The accumulation of ammonia can significantly exacerbate cellular oxidative stress. On one hand, ammonia increases ROS levels by inhibiting the activity of key antioxidant enzymes. Superoxide dismutase (SOD) is the first line of defense against superoxide anions, and studies have shown that high concentrations of ammonia inhibit SOD activity, leading to impaired clearance of superoxide anions and elevated ROS levels. On the other hand, as previously mentioned, ammonia-induced mitochondrial dysfunction is itself a major source of ROS production. Damaged electron transport chains leak electrons, directly leading to ROS generation (57, 58). This ammonia-driven accumulation of ROS is not a unidirectional process but forms a vicious positive feedback loop. ROS can damage organelles and biomacromolecules, potentially further exacerbating protein catabolism, thereby producing more ammonia. Furthermore, ROS, as an important signaling molecule, can activate various pro-carcinogenic signaling pathways, including NF-κB, which in turn may regulate the expression of metabolic enzymes, affecting ammonia production and metabolism. This “ammonia-ROS” cycle collectively drives the proliferation of tumor cells, DNA damage, genomic instability, and metastatic potential (57, 59).

### 6.2 Crosstalk between ammonia and hypoxia signals

Hypoxia is another common feature of the solid TME, initiating an adaptive response centered around HIF-1α. There exists a close bidirectional regulatory relationship between ammonia signals and hypoxia signals. Firstly, ammonia can enhance hypoxia signals. In a high-ammonia environment, the stability of HIF-1α protein within cells is increased (25). This may be due to ammonia altering the intracellular metabolic state (such as the levels of TCA cycle intermediates) or pH, thereby affecting the activity of HIF-1α degradation-related enzymes like prolyl hydroxylases (PHDs). The stabilized HIF-1α translocates to the nucleus, initiating the transcription of a series of downstream hypoxia response genes. The proteins encoded by these genes are involved in glycolysis, angiogenesis, cell survival, and invasion, thereby enhancing the adaptability of tumor cells in low-oxygen environments (60).

Moreover, hypoxic signals can also reciprocally regulate ammonia metabolism. In a hypoxic environment, the activation of HIF-1α can upregulate the expression of enzymes related to ammonia metabolism, particularly GLS (61). The increased expression of GLS indicates an enhanced capacity of cells to process glutamine and generate ammonia. This not only provides an alternative energy source for cells under hypoxia (through the breakdown of glutamine into the TCA cycle) but may also further stabilize HIF-1α by producing more ammonia, forming another positive feedback regulatory loop (62). The cross-regulation between ammonia and hypoxic signals, along with the ammonia-ROS cycle, constitutes a highly integrated stress response system. It enables tumor cells to collaboratively respond to various microenvironmental stresses; for instance, the presence of hypoxia enhances the cells ability to utilize glutamine and process ammonia, while the accumulation of ammonia helps cells better adapt to hypoxia. The resilience of this network explains why treatment strategies targeting a single stress pathway often show limited efficacy and suggests that future approaches may need to adopt systemic combination therapies that simultaneously intervene in tumor metabolism, hypoxia, and redox states.

## 7 Clinical significance and therapeutic approaches

The central role of the ammonia signaling network in tumor biology makes it an attractive target for clinical translation, encompassing various aspects from diagnosis and prognostic assessment to therapeutic intervention. However, the successful application of strategies targeting ammonia metabolism in the clinic hinges on a shift from a one-size-fits-all approach to a biomarker-based precision medicine strategy.

### 7.1 Ammonia signaling network as a source of biomarkers

Numerous studies have confirmed that the levels of ammonia in circulating serum are significantly associated with poor prognosis in various cancers. In tumors such as head and neck squamous cell carcinoma (HNSC) and HCC, patients with elevated blood ammonia levels tend to have later tumor stages, more aggressive disease, and lower overall survival rates. Furthermore, gene characteristics defined by the ammonia-induced cell death (ACD) pattern, known as ACD markers, have also been shown to serve as independent prognostic indicators for predicting patient survival outcomes. These findings suggest that monitoring serum ammonia levels or ACD-related markers in tumor tissues may become effective tools for assessing tumor malignancy and predicting patient prognosis (63–65). The advancement of positron emission tomography (PET) technology has made it possible to non-invasively and dynamically monitor tumor ammonia metabolism. By using radiolabeled ammonia as a tracer, it allows for real-time, quantitative assessment of ammonia uptake and metabolic flux in tumor tissues. Preliminary clinical studies, particularly in HCC, have already demonstrated its potential. Research has found that baseline ammonia uptake kinetic parameters of tumor lesions may predict early disease progression, while changes in ammonia metabolism in non-tumor liver tissues after treatment are associated with long-term survival of patients. Although the current ability of this technology to distinguish treatment responses is still limited, it provides a new dimension for early diagnosis, assessment of tumor heterogeneity, and monitoring treatment responses. In the future, it is expected to be combined with other biomarkers such as genomics to guide personalized treatment (66, 67).

### 7.2 Therapeutic strategies targeting ammonia signaling networks

Ammonia scavengers represent the most direct strategy aimed at reducing systemic or localized ammonia burden. Drugs such as Sodium Benzoate and Sodium Phenylbutyrate work by providing alternative nitrogen clearance pathways, binding to glycine and glutamine respectively, to form products that can be excreted via urine, thereby effectively lowering blood ammonia levels. Preclinical studies, particularly in HCC models, have confirmed that Sodium Benzoate exhibits significant antitumor activity, capable of inducing apoptosis, inhibiting the cell cycle, and modulating key signaling pathways such as PI3K-AKT (68, 69). Although these drugs have been applied in the treatment of hyperammonemia due to urea cycle disorders, their exact efficacy and safety in cancer treatment still require validation through larger-scale clinical trials. Given the critical role of ammonia transport proteins in maintaining ammonia homeostasis in tumor cells, inhibiting their function has emerged as a novel strategy. Notably, SLC4A11 has become an appealing target after being identified as a key ammonia influx conduit in HCC cancer stem cells (19). Specifically inhibiting the function of SLC4A11 can disrupt CSCs utilization of ammonia in the microenvironment, depriving them of this crucial nitrogen source and thereby inhibiting their stemness maintenance and tumor-initiating capabilities. This represents a highly targeted approach towards CSCs with tremendous therapeutic potential. Moreover, inhibitors targeting Rh glycoproteins are also under exploration, although related research is still in its early stages (18).

**Figure 4:**
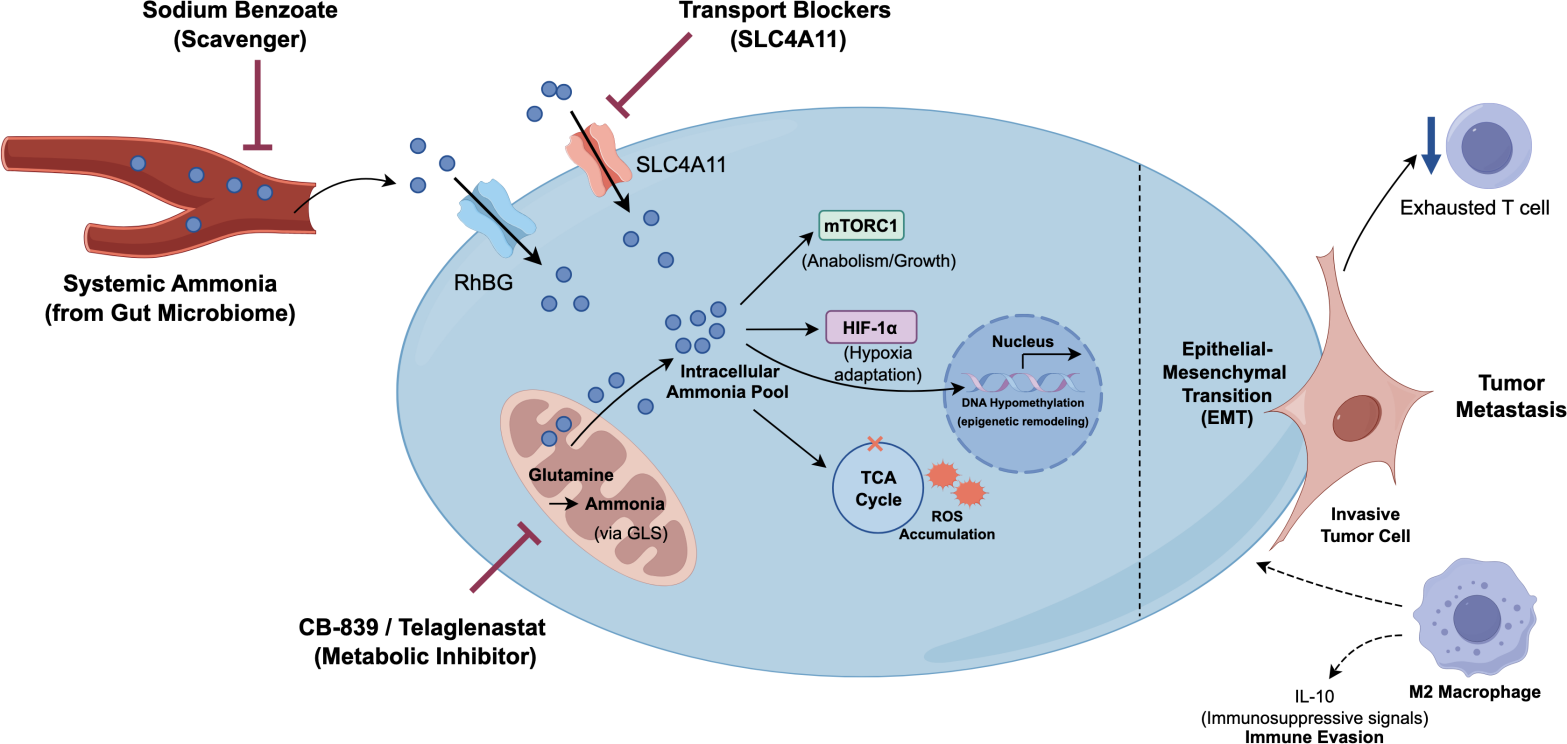
A comprehensive regulatory landscape of the ammonia signaling network and targeted therapeutic strategies.

Inhibition of glutamine metabolism is currently the most extensively researched strategy that has entered clinical trials. Its core principle is to block the conversion of glutamine to glutamate and ammonia by inhibiting GLS, thereby reducing ammonia production at its source. Telaglenastat (CB-839) is the first-in-class oral GLS inhibitor that has undergone extensive preclinical and clinical studies across various solid tumors and hematological malignancies. The mixed results from early clinical trials, most notably the failure to meet the primary endpoint of improving progression-free survival in the Phase II CANTATA study for advanced renal cell carcinoma, highlight a critical translational hurdle: not all tumors equally rely on glutamine metabolism (70). The observed limitations in efficacy within these unselected cohorts are largely attributed to the profound metabolic plasticity of tumor cells. Upon GLS inhibition, resilient tumors rapidly activate alternative survival mechanisms. For instance, they may upregulate compensatory asparagine synthesis, enhance the utilization of glucose-derived carbon, or exploit alternative extracellular nitrogen sources, thereby circumventing the intended metabolic blockade and rendering monotherapy ineffective (71). This has prompted the development of biomarker-based patient stratification strategies. A substantial body of preclinical evidence and subsequent clinical trial designs, such as the BeGIN study (NCI-2019-01365), indicate specific genetic mutations that can lead to a dependence on the glutamine pathway characterized by synthetic lethality. In tumors such as non-small cell lung cancer (NSCLC), inactivation mutations of the KEAP1 gene or activation mutations of NRF2 (encoded by the NFE2L2 gene) lead to persistent activation of the NRF2 signaling pathway. NRF2 is the principal regulator of cellular antioxidant stress, and its excessive activation drives the synthesis of large amounts of glutathione (GSH) to counteract oxidative stress. Since the synthesis of GSH requires glutamate as a precursor, this creates a significant dependency of these tumor cells on glutamate generated from glutamine (72–75). Therefore, in tumors with KEAP1/NRF2 mutations, the use of GLS inhibitors (such as Telaglenastat) can effectively disrupt the supply of glutamate, leading to GSH depletion and oxidative stress catastrophe, thereby selectively killing tumor cells (https://www.clinicaltrials.gov/study/NCT04265534). In addition to KEAP1/NRF2, other genetic backgrounds may also predict sensitivity to GLS inhibitors. For instance, in patient-derived xenograft (PDX) models of colorectal cancer with PIK3CA mutations, CB-839 demonstrated monotherapy efficacy and synergized with 5-fluorouracil (5-FU) to induce tumor regression. This series of research endeavors clearly indicates that the future of targeted ammonia/glutamine metabolism lies in precision medicine (76). It is essential to utilize genomic biomarkers (such as the KEAP1/NRF2 mutation status) and potential metabolic imaging biomarkers to accurately identify patient subgroups whose tumor biology is truly hijacked by this metabolic pathway, thereby maximizing therapeutic efficacy (77). The following two tables summarize the multifaceted roles of the ammonia signaling network in tumor progression, as well as specific therapeutic strategies targeting this network (**Tables 2**, **3**). A comprehensive visual integration of ammonia sources, core molecular pathways, pathological outcomes, and corresponding clinical interventions is presented in **Figure 4**, offering a holistic perspective of the entire regulatory network.

**Table 2:** Multiple roles of ammonia signaling network in tumor progression.

Biological process	Key mechanism	Impact on tumor	Key molecular targets
Metabolic Reprogramming	α-KGDH inhibition; SREBP-1 activation	Strengthens Warburg effect; promotes lipid synthesis	α-KGDH, SREBP-1
Epigenetic Reprogramming	SAM depletion; KDM inhibition	Global DNA hypomethylation; increased histone methylation	DNMTs, KDMs
Tumor Metastasis	TGF-β/Smad pathway activation	Induces EMT, enhances invasive ability	TGFβR, ZEB1/2
Immune Evasion	Inhibits perforin; induces T cell death; M2 macrophage polarization	Weakens cytotoxicity; clears immune effector cells; creates immune-suppressive environment	GLS, ammonia transporter proteins

**Table 3:** Targeted treatment strategies for ammonia signaling network.

Treatment strategy	Drug/category	Mechanism of action	Key findings	Potential stratification biomarkers
Ammonia Clearance	Sodium Benzoate	Provides alternative nitrogen clearance pathway	Demonstrates anti-tumor activity in HCC preclinical models	Urea cycle defects; high blood ammonia
Transport Inhibition	SLC4A11 Inhibitors	Blocks ammonia influx in HCC cancer stem cells	Inhibits tumor stemness and reduces tumor initiation	High expression of SLC4A11
Metabolic Inhibition	Telaglenastat (CB-839)	Inhibits GLS	Effective in certain genetic backgrounds; synergizes with chemotherapy/other targeted drugs	KEAP1/NRF2 mutations; PIK3CA mutations

## 8 Future outlook

Research on ammonia signaling networks has made significant progress, yet it remains in a phase filled with opportunities and challenges. Future studies need to integrate cutting-edge technologies and concepts from multiple disciplines to deeply understand the complexities of this network from a systemic perspective, ultimately translating these insights into effective clinical practices.

### 8.1 Technological frontiers: dynamic monitoring and advanced models

Current research predominantly relies on static endpoint measurements, making it challenging to capture the spatiotemporal dynamics of ammonia in the TME. Developing technologies that can monitor ammonia concentration in real-time and with high resolution within the TME is crucial. Novel sensors based on electronic noses and intelligent algorithms provide possibilities for dynamic monitoring in *in vitro* and animal models. At the clinical level, further optimization of imaging techniques to enhance their sensitivity and specificity will aid in achieving precise, non-invasive assessments of ammonia metabolism (67). Concurrently, the application of single-cell multi-omics (such as scRNA-seq and scATAC-seq) and spatial transcriptomics technologies will enable us to dissect the heterogeneity within tumors at an unprecedented resolution. These technologies can reveal differences in ammonia metabolism among various tumor cell subpopulations (such as cancer stem cells and invasive front cells) as well as between different stroma and immune cells, thereby allowing for a more accurate understanding of the role of ammonia signaling in intercellular communication within the TME. Furthermore, more clinically relevant preclinical models, such as patient-derived organoids (PDOs) and PDXs, are essential for evaluating the efficacy of targeted ammonia signaling drugs and screening sensitive patients. These models retain the genetic and phenotypic heterogeneity of the original tumors, enabling better predictions of drug responses in humans and serving as an important bridge between basic research and clinical trials.

### 8.2 The necessity of integrating systems biology

The ammonia signaling network is a complex system, whose effects arise from the interactions of multiple pathways. Future research must adopt the principles of systems biology, integrating multi-dimensional data such as metabolomics, epigenomics, transcriptomics, and proteomics. By constructing computational models such as genome-scale metabolic models (GSMMs), it is possible to simulate the flux changes in ammonia metabolism under different genetic backgrounds and microenvironmental conditions, predict key metabolic nodes and drug targets, and reveal the synthetic lethal vulnerabilities associated with ammonia or glutamine addiction. This integrated analysis will aid in understanding the metabolic resilience of tumors from a network perspective and designing more intelligent therapeutic strategies (48).

### 8.3 Revealing the role of the gut microbiome

As a major source of systemic ammonia, the role of the gut microbiome in tumor ammonia metabolism and therapeutic response is an emerging field that requires exploration (9). The composition and function of the microbiome can directly affect the host s nitrogen metabolic balance and blood ammonia levels, potentially regulating ammonia concentrations in the TME (11). Furthermore, the microbiome itself is a crucial factor in modulating the host immune system, particularly in response to immune checkpoint inhibitor (ICI) therapy. Therefore, investigating the interactions among the gut microbiome, ammonia metabolism, and tumor immunity may provide new insights for enhancing anti-tumor therapies, including those targeting ammonia metabolism and immunotherapy, through the modulation of gut microbiota.

### 8.4 Overcoming treatment resistance and designing rational combinations

Drugs targeting a single metabolic pathway often face the challenge of acquired resistance. Tumor cells can evade the cytotoxic effects of glutamine metabolism inhibitors through various mechanisms. For instance, they may upregulate GLUL to facilitate *de novo* glutamine synthesis or switch to alternative amino acids, such as aspartate and branched-chain amino acids, to create metabolic bypasses. Understanding these resistance mechanisms is a prerequisite for developing next-generation therapies and designing effective combination treatment regimens.

Through a systematic analysis of the multifaceted functions of ammonia signaling networks, various synergistic therapeutic strategies can be constructed. Firstly, in the combined application of immune regulation and metabolic intervention, considering the significant suppressive effect of ammonia on immune function, the combination of GLS inhibitors with immune checkpoint inhibitors (such as PD-1/PD-L1 antibodies) can achieve dual objectives: on one hand, directly inhibiting tumor proliferation, and on the other hand, alleviating the immunosuppressive state of the TME, thereby enhancing T cell anti-tumor activity and producing synergistic therapeutic effects (78). Secondly, in the synergistic strategy of metabolism and epigenetic regulation, based on the bridging role of ammonia between the two, the combination of GLS inhibitors with epigenetic modulators (such as DNMT or HDAC inhibitors) may synergistically correct the abnormal gene expression patterns in tumors. Furthermore, multi-target metabolic intervention strategies that simultaneously inhibit ammonia metabolic pathways and other key metabolic pathways (such as glycolysis or the mTOR signaling pathway) may induce a “synthetic lethality” effect, enhancing the cytotoxic effects on tumor cells. Currently, clinical trials combining GLS inhibitors with mTOR inhibitors for KEAP1-mutant tumors are based on this theoretical foundation (79). However, the clinical application of metabolic inhibitors still faces numerous challenges, including optimizing dosing regimens to balance tumor suppression with toxicity to normal tissues (especially immune cells and intestinal epithelial cells), developing reliable biomarkers for precise patient selection, and designing effective clinical trial endpoints to assess the efficacy of metabolic-targeted therapies. Addressing these issues requires collaborative innovation across the fields of basic research, clinical practice, and drug development.

## 9 Conclusion

The ammonia signaling network serves as a core regulatory hub for the malignant progression of tumors, providing a novel and integrative paradigm for our understanding of tumor biology. This review systematically elucidates how this network acts as a critical node connecting tumor cell metabolism, epigenetic regulation, and metastatic mechanisms. The research not only reveals how tumor cells cleverly adapt to and remodel their microenvironment by regulating ammonia metabolism but also clarifies the crucial functions of ammonia signaling in epigenetic reprogramming and immune evasion. As research on the ammonia signaling network deepens, we anticipate uncovering its specific mechanistic roles at different stages of tumor metastasis, which will lay a solid theoretical foundation for developing innovative intervention strategies. Despite current studies indicating that therapeutic approaches targeting the ammonia signaling network, such as ammonia scavengers, transport inhibitors, and glutaminase inhibitors, hold significant anti-tumor potential, there are still substantial challenges in successfully translating these basic research findings into clinical applications. Firstly, the complexity and context dependency of the ammonia signaling network necessitate caution when interpreting different experimental results. In certain tumor types or microenvironments, enhanced ammonia metabolism may promote tumor proliferation, while in others, it may exert inhibitory effects. Therefore, conducting in-depth and meticulous differential studies on the mechanisms of the ammonia signaling network in various tumors and their specific microenvironments is crucial.

Future research should focus on developing more precise intervention strategies to fully unleash the therapeutic potential of targeted ammonia signaling networks. By thoroughly investigating the role of ammonia signaling at various stages of tumor progression, as well as its dynamic interactions with other metabolic pathways and signaling mechanisms, researchers can identify the most critical therapeutic targets and design more effective treatment regimens. Furthermore, exploring strategies that synergistically apply ammonia-targeted therapies with immunotherapy, chemotherapy, and other targeted treatments will open up promising new avenues for anti-tumor therapy. It must be recognized that research on ammonia signaling networks is still in its early developmental stages, and the realization of its clinical applications requires robust support from further basic and clinical studies. Researchers should strengthen multi-center collaborations to promote the standardization and systematization of ammonia signaling-related research, enabling more effective validation of its efficacy and safety in clinical practice. Additionally, research designs must adequately consider patient heterogeneity to advance the goals of precision medicine. In summary, the exploration of ammonia signaling networks provides groundbreaking insights into tumor metabolic dysregulation, epigenetic variations, and metastatic processes, holding significant theoretical value and clinical significance. With the continued advancement of research in this field, we have reason to believe that its application in cancer treatment will achieve significant breakthroughs, ultimately providing more effective and personalized treatment options for a wide range of cancer patients.
